# Progress in Obstetrics and Gynecology1The second series of Harrington Lectures delivered at the University of Buffalo, May 24, 25, 26, 1910.

**Published:** 1910-09

**Authors:** J. Clarence Webster

**Affiliations:** Chicago Ill.; Professor of Obstetrics and Gynecology in Rush Medical College, affiliated with the University of Chicago


### BUFFALO MEDICAL JOURNAL

Vol. LXVI. SEPTEMBER, 1910. No. 2

### ORIGINAL COMMUNICATIONS

### Progress in Obstetrics and Gynecology[1]

By J. CLARENCE WEBSTER, M.D.
Chicago Ill.
Professor of Obstetrics and Gynecology in Rush Medical College, affiliated with the University of Chicago.

Lecture II.—Obstetrics—Part I.

AMONG the means of checking post-partum hemorrhage is mentioned careful bandaging of the abdomen in order to press the uterus well into the pelvis, thus compressing blood-vessels.

After difficult or tedious labors, it was advised to envelop the loins of the patient in the raw skin of a sheep flayed alive, and to place on her abdomen the skin of a hare flayed alive, and washed in the hot blood of the animal. These applications lasted two hours in winter and one in summer. In France this was a common practice and is mentioned in particular in Louyse Bourgeois's book. According to her "it constringes the parts over-stretched by labor, removes bad blood and chases away the vapors."

In some cases the afterbirth was placed on the abdomen instead of a hare's skin. Louyse states that the sheep must be a black one, and Ambrose Paré, in his work, declared that a white sheepskin was not efficacious.

An interesting story is told of a birth in the Royal Family, at which the black sheep was used on the mother. The unfortunate animal, which had been flayed alive in the room next to the delivery chamber, rushed after the butcher who had hastily removed its skin and had hurried to the bedside leaving the door open behind him. There was a sudden scattering of the lords and ladies in attendance.

For delayed labors due to rigidity of the cervix powdered hellebore or pepper was blown into the nostrils of the mother in order to make her sneeze as much as possible. Several drugs

1. The second series of Harrington Lectures delivered at the University of Buffalo, May 24, 25, 26, 1910.

were also given, and the milk of another woman, if it could be obtained, was mixed with maidenhair and applied warm to the navel.

Throughout the book prescriptions of the most complicated nature are used. As an example one which was used in delayed labors was the following:

> Take two yolkes of eggs and boyle them in ould wine, then mix with them these spices; cinnamon half an ounce, rind of cassia two drams; or you may leave out the cassia, and instead thereof put in the more cinnamon; saffron half a scruple, savine, betonie, Venus-haire, dittaine, fenugrekke, lawrelberries, mint, of each one dram; the bone of the heart of a hart, pearles prepared, mingle all these with sugar, and make a thick powder and give it.

The danger of leaving any part of the placenta or membranes in the uterus is clearly pointed out in the following terms: "retention causes suffocation and divers other evils, for being long detained, they putrefie and cause an evil smell, which ascending up to the heart, liver, stomach, diaphragma, and so to the brain, cause pains in the head and the lungs, shortness of breath, faintness, cold sweats; so that there is great danger." The chapters dealing with hemorrhage in connection with labor, show that the writers were alive to the importance of careful and active treatment.

The advice that is given regarding the choice of a wet nurse contains the following sentences: "see whether her sight be no way imperfect, as whether she be squint-eyed or have a downcast look; you must have a special care that she be not red haired, for their milk is extremely hot.

Among the causes of barrenness are mentioned: "against which it is affirmed that the drinking a draught of cold water that drops from the mouth of a young horse as he drinks is very potent." The following are also mentioned: "all sorrow, anger and much sleep; the eating of milk, fresh cheese, and anything that is made of dough." Numerous other articles of diet are to be avoided, for example, spinach, beets, lettuce, nuts, cherries, onions. All these are apt to give the womb "some cold and moist distemper, whereby it cannot draw into itself the semen."

In the final chapter on the instructions of a dying midwife to her daughter, some excellent advice is given, for example:

> Be diligent, and leave nothing unsearched that may tend to the advantage of thy practice. And to this end be always learning to the last day of your life; which that thou mayest not cease to do, be always humble . . . . Hide none of those good receits which thou knowest, either from midwives or physicians; for otherwise they will estiem them as little as those of mountebanks. . . . . Above all you must be aware (for any treasure in the world) of adhering to one vice, such as they are guilty of who give remedies to cause abortion. . . . If you are sent for to any house, inform yourself of what condition they are; and whether they be rich, or whether they be the poorest creatures in the world, serve them with like pains and affection.

Never be dismayed if everything go not well, for fear disorders the senses; and a person that keeps her wits together, without suffering them to be scattered by fear, is capable of giving assistance in weighty affairs, and especially where things are done with leisure; for in some cases nature helps us marvelously when we are most at a stand. . . . My last advice is that thou do well, and in so doing fear nothing but God that he may bless thee and thy endeavors.

Gradually popular opposition to the practice of obstetrics by men died down and the term man-midwife (*accoucheur*) became applied to those who were expert in this branch of work. Dionis writing in 1718 in reference to the qualifications necessary in such a practitioner says:

In a word, he must show himself a perfect honest man, who squares all his actions by the word of God. He must therefore, be virtuous, of a sweet temper, affable, full of compassion, and always contented with any handsome or moderate fee that is given him.

He begins his description of the necessary qualifications of a midwife thus:

Midwives ought not only to have all the good qualities required in men-midwives, but must also leave off several vices proper to their sex and profession.

Here is the account given by Mr. Tolver, a London man-midwife, who visited Paris after the middle of the eighteenth century, of the course of lectures given by a M. Payen:

This profession has rose into notice, rather through intrigue than merit, and was set up in opposition to M. Levret. The lectures he reads were penned by a very eminent physician and man-midwife, expressly for that purpose. . . . Each course continues about three or four months; and as the expense is only one guinea, the pupils of both sexes are seldom less than threescore. Here barbers, women, and regulars promiscuously assemble, and are present together upon all occasions, a circumstance very disgusting to the gentleman, and frequently repugnant to the delicacy of a Briton.

There were various reasons for the gradual transfer of obstetric practice from midwives to physicians. Prominent among these was the renaissance of anatomical investigations after the fifteenth century. Previous to this time the anatomy of the human body was very imperfectly known, and was mostly based on the writings of the Ancient Greek authors, the oldest of which are those of Hippocrates and Aristotle, who described the human uterus as it was found in animals. Though human dissection had been practised in Alexandria by Herophilus and Erasistratus, it left no permanent impress on medical literature. Even the celebrated Celsus had no accurate knowledge of the female genitalia. Diocles described mammary processes in the uterus used by the fetus for its nourishment.

Soranus in the beginning of the Christian era described accurately the relation of the uterus to the bladder, the uterine ligaments, the ovarian arteries, arbor vitæ and external genitals. He missed the fallopian tubes, however. Galen used the Hippocratic description of the uterus, stating that he intended to write on the function of its two horns. Mondino, of Bologna, in the fourteenth century dissected the female cadaver, but he described the uterus as having seven cavities.

The sixteenth century marks the real commencement of anatomical study, the following workers being the chief contributors to our knowledge of female pelvic anatomy previous to the nineteenth century: Berengario da Carpi (1522), Vesalius (1543), Eustachius (1552), Fallopius (1561), De Graaf (1672), Malpighi (1687). In the eighteenth century Ruysch, Douglas, Haller, Albinus, Smellie, Donald, and Alexander Monro, Roederer, William Hunter.

Albinus was the first to describe the gravid uterus. He published a series of seven plates in Leyden in 1747. Though the drawings were beautiful they were wanting in accuracy. Ruysch worked at the musculature of the pregnant uterus describing the fibers of the fundus as of use in expelling the placenta.

Smellie in 1754 and Roederer in 1757 published plates of the gravid uterus. These and the publication of Albinus are to be regarded as the first contributions to the literature of the anatomy of pregnancy.

In 1774 William Hunter published his "Atlas on the Anatomy of the Human Gravid Uterus Exhibited in Figures." He died before his description of the plates appeared, but his manuscript was issued and edited by Baillie in 1794. In the nineteenth century the anatomy of pregnancy and labor was further elaborated both by dissectional and sectional studies.

Though in previous centuries Leonardo da Vinci Berengario da Carpi, Vesalius, Eustachius, Ambrose Paré and others gave some attention to the relationships of structures, they made no special study of the female pelvis. This was first described in sections by Huschke in 1844. Freezing, first employed by Pirogoff in making topographical studies, has been used to great advantage in Germany and Scotland especially in the study of the anatomy of pregnancy, labor and the puerperium. In the latter part of the nineteenth century and the beginning of the twentieth, the most careful histologic studies of the female genitalia were made both in the non-pregnant condition, in pregnancy, labor and the puerperium, so that, at the present time, it may be stated that very little remains for investigators to discover in this field of work.

Another great reason for the transfer of obstetric practice from midwives to men was the invention of forceps. Though this instrument was kept a secret in the Chamberlen family in England during the latter part of the seventeenth century, it gradually came into use in Europe during the eighteenth century. It is on record that the Amsterdam Medico-Pharmaceutic College in the first half of the eighteenth century would give no license to one who could not use the forceps (or rather lever).

After the important improvements introduced by Levret (1751), and Smellie (1752), the forceps became widely known, and as the public realised that living children could be produced in cases where previously the mother might die or the infants be destroyed by embryotomy, men-physicians became a much more important factor in obstetric practice. Education also proved a very important factor in bringing about this change. For centuries the midwives worked empirically to a great extent. They used rules and precepts which for the most part had been transmitted orally from generation to generation.

Before the era of printing there were few books. There was no hospital or dispensary teaching and little individual instruction. The renaissance of anatomical investigation in the sixteenth century not only led to the establishment of a more scientific basis for the study of medicine, but stimulated the ardor and interest of teachers and students in various parts of Europe. As Goodell puts it: "in proportion as people grew wiser by reading books and by having them to read, the ignorance of midwives became more and more manifest. The physician developed with the times the midwife did not. The battle between knowledge and ignorance is never a drawn one; either Christian must die or Apollyon give way."

In the eighteenth century students learned mostly from being apprentices to practitioners. Certain men of prominence gave special instruction, for example, Smellie in London; Levret in

France, and Ruysch, professor of anatomy in Amsterdam, who sold a large anatomical collection to Peter the Great, taught obstetrics for a time. The first university professorship of obstetrics and diseases of women was established in Edinburg in 1726, Joseph Gibson being professor. A generation later the first German chair was established in Göttingen, Roederer being the professor.

In Italy King Victor Amadeus II. of Piedmont in 1728, set apart a ward in San Giovanni Hospital for lying-in women where midwives could be taught. At Bologna, Galli, Professor of Surgery during the first part of the eighteenth century was made professor of obstetrics in 1757, as he had been teaching the subject privately for some time.

During the nineteenth century great advances were made, dispensaries for women, special obstetric wards in general hospitals, and independent maternities being everywhere established. Until the epoch-making researches of Pasteur and Lister, the value of these institutions was greatly marred by the great prevalence of septic infection. Now that this terror has been overcome, hospital obstetric practice has been established as the safest and most satisfactory. Indeed, at the present time. in the leading civilised countries, private obstetric practice cannot compete with institutional practice as regards freedom from mortality and morbidity. Various prominent authorities, especially in England and America, have recently drawn attention to this state of matters, criticising many of the mistakes commonly made in general practice.

In conclusion I wish to refer to three topics of great importance to the general practitioner—namely, douching of the genital tract, digital examination, and instrumental delivery. Too strong a protest cannot be urged against the indiscriminate and unscientific use of antiseptic vaginal douches in obstetric practice. In spite of the careful work carried on in different countries by numerous observers, there is widespread ignorance of the following facts—namely, that there is a normal bactericidal influence exerted in the genital canal tending to produce continued asepticity, and that this influence may be considerably weakened by the chemical action of many antiseptics. Many physicians are accustomed to employ antiseptic douches in pregnancy as a prophylactic measure. That this is unnecessary in the great majority of instances has been sufficiently demonstrated. Of course, if there is any local acute or chronic infective or venereal process on vulva, vagina or cervix, the pregnant woman should be treated most carefully so as to diminish the chance of infection in labor, and to achieve this end the most vigorous use of antiseptics may be necessary.

Apart from such conditions, douches are unnecessary in pregnancy. It is scarcely necessary to remark in this connection that the condition of the husband is of importance. Sexual intercourse should not be allowed during the weeks previous to labor, and if he shows evidences of uncured venereal disease coitus should be prohibited for months.

There has been much difference of opinion among workers as to the normal condition of the vaginal contents, but the causes of their differences have been recently fairly well elucidated, and at the present time it may be accepted as proven that the genital canal tends to be maintained in a state of asepticity by natural means. Prophylactic douching during pregnancy is unnecessary, neither is it needed after labor, if the patient be not exposed to contamination by those who attend her during parturition. Given a sterile genital canal, the aim should always be to conduct the labor aseptically.

Most frequently the sterile tract is contaminated by unnecessary or careless manipulation carried out by doctor and nurse. Nothing is more to be deprecated than frequent routine vaginal examination of parturient women in practice. It is absolutely unnecessary in the great majority of cases. The abdominal examination is ordinarily a sufficient source of information to the physician, though there are, of course, occasions when vaginal examination is necessary. Then it should be made with as much care as is exercised in the best technic of surgical operations, not in the hasty or careless manner only too frequent in ordinary practice. There should be as much strictness as regards cleanliness, in introducing the fingers into the vagina of the parturient women, as in exploring the peritoneal cavity in an abdominal section. In the latter proceeding every precaution is taken to cleanse the skin of the patient, as well as that of the operator's hands. How often, in obstetrical practice, is the accoucheur satisfied with a hurried application of soap and water, a momentary dip in some antiseptic solution, often not of measured strength, but made of an unknown number of drops in an unknown quantity of water? How often does he neglect to cleanse the external parts with the same thoroughness which he observes in preparing for surgical work?

The hair-covered vulva is ordinarily infested with myriads of microörganisms, including many of a pathogenic nature. It matters little how clean the fingers are, if this region be not attended to. They cannot be introduced into the vagina without carrying the external contamination. The only way in which this risk can be reduced to a minimum is by shaving the vulva before every labor, cleansing it thoroughly when labor begins, and keeping it covered during labor with dressings soaked in an antiseptic solution. If shaving cannot be carried out, the hairs may be clipped close with scissors.

As regards hands, I believe, that in addition to careful cleansing, sterile rubber gloves should always be used when examinations or operative procedure is to be carried out. As regards after-douching, in the majority of cases it is not necessary. When there is a tendency to the accumulation of blood clots in the vagina, sterile salt solution may be used. Antiseptic solutions are only to be employed when local infection exists before labor, when the patient is subjected to the risk of infection during labor or when the perineum has been badly lacerated. To keep the external parts as clean as possible, it is advisable to wash them with an antiseptic lotion during the first few days of the puerperium or to keep them covered with dressings soaked in the solution.

*The Abuse and Misuse of Forceps.*—There can be little doubt that since the introduction of anesthesia into obstetrical practice artificial delivery by various procedures has become very much more frequent than in the pre-anesthetic days, when interference was avoided as much as possible because of the increased suffering to the patient. Much of the newer practice is legitimate and life-saving, but it cannot be denied that anesthesia has also served as a cloak to widespread and unwarrantable license in the employment of methods for hastening labor.

It is too often forgotten that the majority of labors are natural, that they should be interfered with as little as possible, that interference, even by the most skilled hands, is always accompanied with risks. It is amazing to notice with what boldness many physicians will undertake the most difficult obstetric maneuvers, when they will positively shrink from performing some surgical operation, which they may have witnessed many times. When it happens, as is not infrequent, that this obstetrical boldness is accompanied with surgical uncleanliness, the result of the conjunction is sometimes the death of the unfortunate patient, often the establishment of a pathological museum in her pelvis. The best evidence of the truth of what I state is derived from our gynecological case records.

Forceps operations, especially, are undoubtedly undertaken with too great frequency. It is common to meet those who use instruments in 10, 20 or 30 per cent. of their deliveries, or even in a larger proportion, so frequent are the indications for interference in their practice. How often these indications are their own creation they do not state. Nor do they confess how frequently they are influenced by a desire to curtail the dreary time of waiting at the bedside, or are inspired by a humane desire to cut short the sufferings of the patient.

To give an idea of the frequency with which forceps are used in various European hospitals, I quote the following list, compiled by Wahl: von Walla, Budapest, 1882-1895, 1.04 per cent.; Kezmarszky, Budapest, 1874-1882, 1.4 per cent.; Abegg, Danzig, 1872-1885, 2.2 per cent.; Leopold, Dresden, 1879-1885, 2.56 per cent.; Gusserow, Berlin, 1882-1886, 2.66 per cent.; Ahlfeld, Marburg, 1881-1888, 3.5 per cent.; von Rosthorn, Prag, 1891-1894, 3.63 per cent.; Braun, Wien, 4.3 per cent.; Fehling, Basel, 1887-1893, 5.33 per cent.; von Säxinger, Tübingen, 6.5 per cent.; Schauta, Innsbruck, 1881-1887, 9.16, per cent.; Schultze, Jena, 11.6; von Winckel, München, 1884-1890, 22.6 per cent.

These figures taken from teaching institutions represent a higher percentage of indications than actually existed, for in many instances the instruments were used, where they were not necessary, in order to give instruction to students. Taking this into consideration, it is indeed, striking that the percentages are so low. As more difficult cases occur in maternity practice than in private, it is very evident that the necessity for instrumental delivery in private practice must be very small.

The risks attendant upon the use of instruments are well recognised by those who are experts in applying them, and who cannot avoid them in a considerable percentage of cases, even by the exercise of marked skill and judgment. Too frequently among those who are not experts and do not work with scientific knowledge, is found a disregard of these dangers. One occasionally hears experienced practitioners boast of the hundreds of women they have delivered without mortality. They do not think of the stretching and tearing which they do not see, and make light of those which are visible, unless of the most severe nature. As long as a patient does not die from hemorrhage or sepsis, the labor is considered satisfactory. Responsibility for a long list of mechanical and infective troubles which follow the disregarded lesions is not shared by them.

As a gynecologist, I cannot too strongly emphasize, among the etiological factors concerned in the production of women's diseases, those which arise from injudicious and meddlesome interference with the normal parturient process.

---

AEROTHERAPY.—J. George Sauer, New York, *Medical Record,* August 13, 1910, proposes that we shall sleep in beds that are so arranged that the head projects out of the window in all weathers. This induces good sleep and good health. To secure this he removes the legs of the head of the bed to the middle of the side frames, so that the head may be pushed out of the window, and the window shut down upon the lower portion of the body, the upper part projecting out of the window, well protected by a hood and warm bedding in winter.

